# Beware of Warfarin-Induced Skin Necrosis in the Setting of Heparin-Induced Thrombocytopenia

**DOI:** 10.7759/cureus.8857

**Published:** 2020-06-27

**Authors:** Natasha Dhawan

**Affiliations:** 1 Hematology and Oncology, Dartmouth-Hitchcock Medical Center, Lebanon, USA

**Keywords:** warfarin-induced skin necrosis, thrombosis, skin necrosis, drug toxicity, heparin-induced thrombocytopenia

## Abstract

Heparin-induced thrombocytopenia (HIT) and thrombosis affect a small minority of patients exposed to heparin. However, given the high proportion of patients who receive heparin during hospitalization, clinicians should be mindful to keep it on their differential. This case involves a 56-year-old woman who developed HIT during a prolonged hospitalization. She was started on a direct thrombin inhibitor, argatroban, until her platelets recovered, was bridged to warfarin, and later developed warfarin-induced skin necrosis (WISN). Patients with prolonged hospitalizations may have an inherent vitamin K deficiency, leading to erratic changes in international normalized ratio (INR). Currently, there are no guidelines to address very high supratherapeutic INR levels in this setting. Prompt diagnosis and close monitoring during treatment are essential to minimize the risk of morbidity and mortality.

## Introduction

Heparin-induced thrombocytopenia (HIT) affects 0.2%-3% of heparin-exposed patients [1]. It can lead to venous and arterial thromboses, though venous thromboses are more common. Treatment involves removing exposure to heparin and starting direct thrombin inhibitors while awaiting platelet count recovery. Once the platelet count has recovered, anticoagulation is traditionally bridged to warfarin. Warfarin-induced skin necrosis (WISN) is a rare complication that occurs in 0.01%-0.1% of patients using warfarin [2]. HIT and protein C deficiency may be risk factors for the development of WISN [3].

## Case presentation

A 56-year-old woman with a history of a deep vein thrombosis during pregnancy and dementia presented with dyspnea and acute hypoxemia. Shortly thereafter, she developed persistent fevers and acute encephalopathy. Initial testing includes lumbar puncture, chest x-ray, multiple blood cultures, urine cultures, respiratory cultures, and rapid plasma reagin for syphilis, all of which were unremarkable. CT of the head was significant for sphenoid sinusitis. The patient was started on meropenem. She was transferred to the ICU for worsening hypoxemia. She developed a non-ST elevation myocardial infarction and lower extremity ultrasound revealed bilateral deep vein thromboses, and she was started on therapeutic heparin.

**Figure 1:**
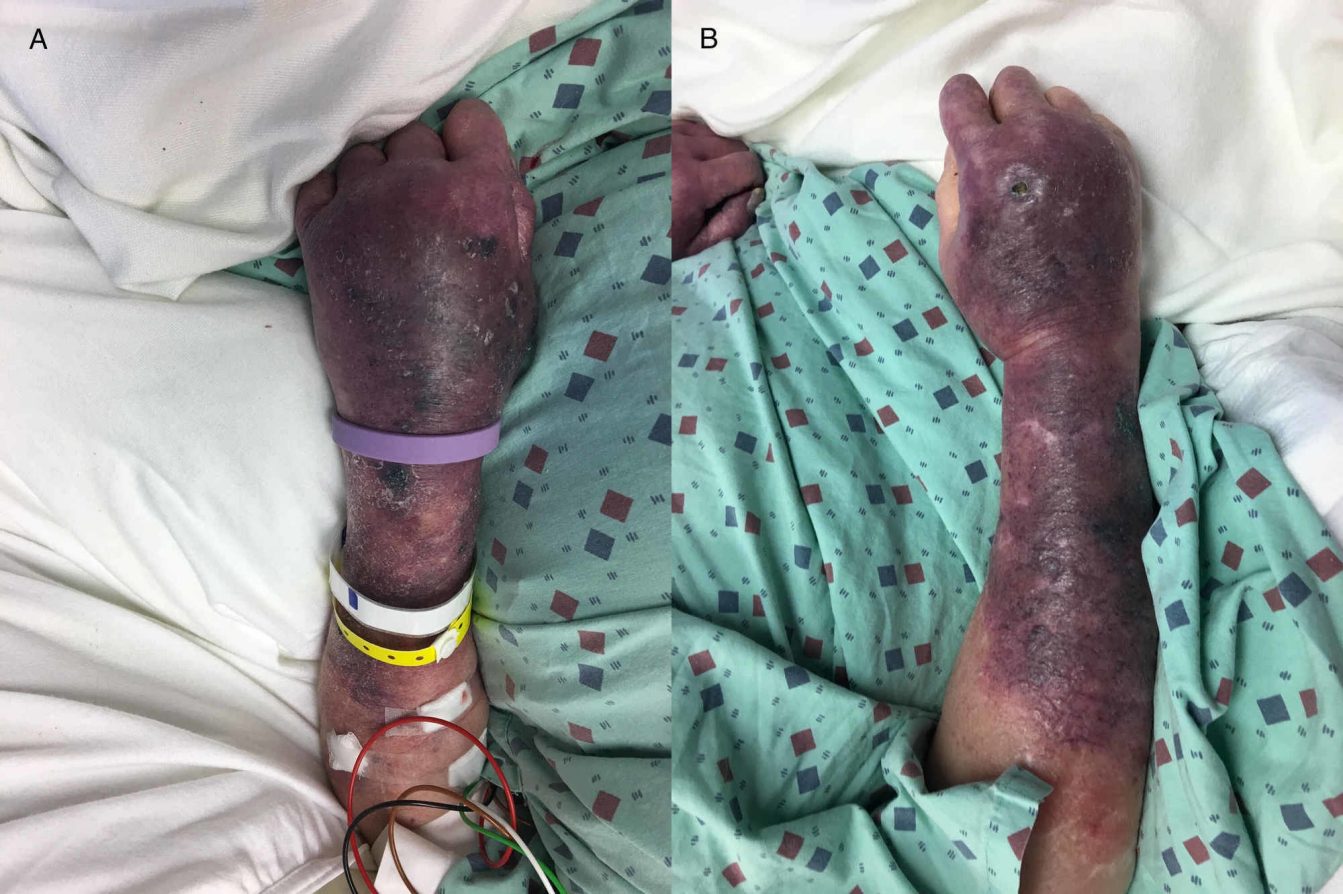
A: Left Upper Extremity. B: Right Upper Extremity

**Figure 2:**
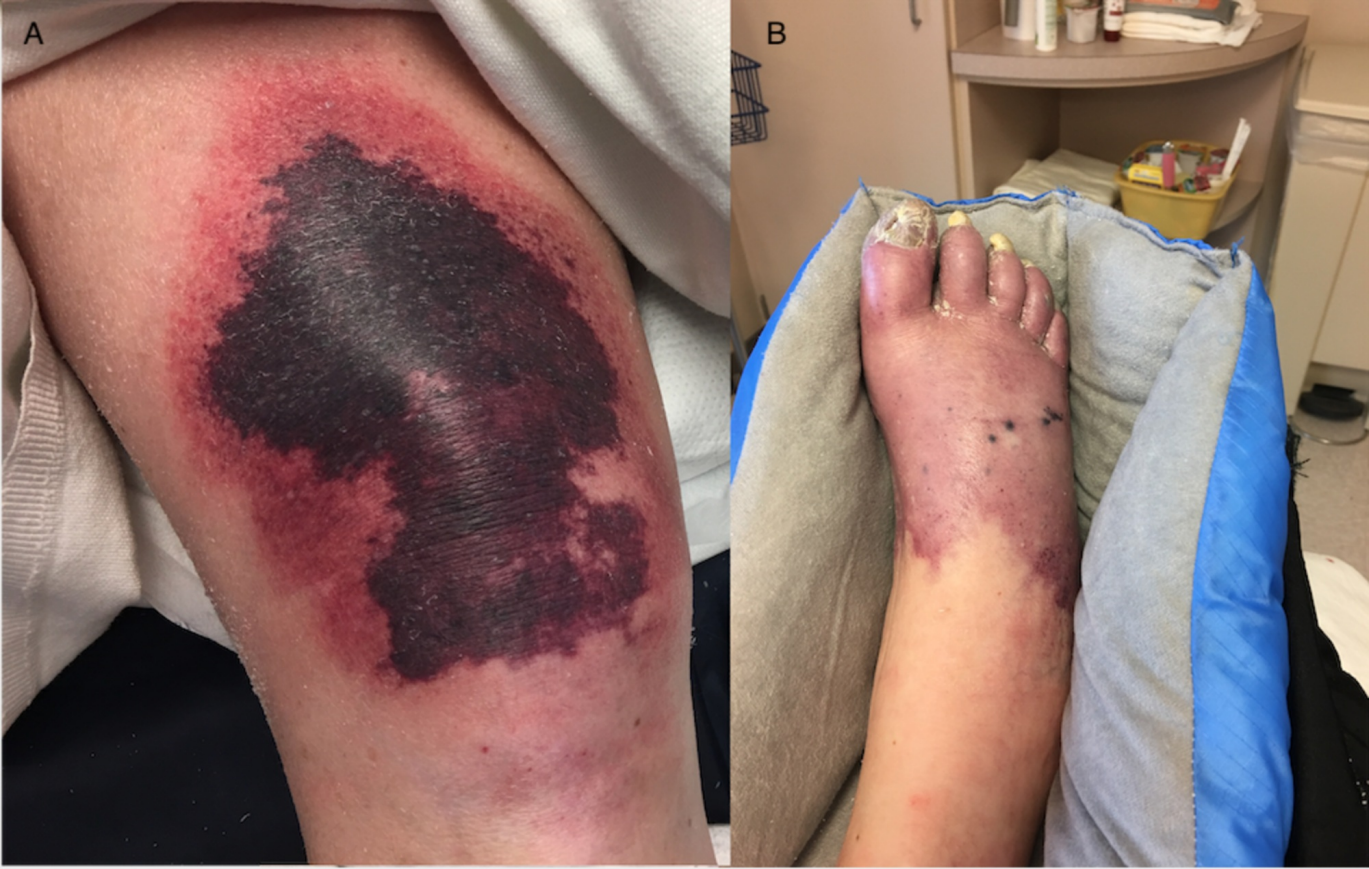
A: Right Knee. B: Right Foot

Over nine days, the patient’s platelet count decreased from 250 to 98 K/µL. Serotonin release assay confirmed HIT. Heparin products were stopped and argatroban was initiated. Her platelet count recovered 16 days later, and she was bridged to warfarin. Her international normalized ratio (INR) increased from 1.91 to 9.39 over the next three days. Argatroban was stopped, repeat INR six hours later revealed an INR of 7.01, and the warfarin dose was held. The following day, the INR was 6.42 and the patient was complaining of right knee pain. She developed purpura over bilateral upper extremities with central necrotic lesions (Figure 1), purpura on her right knee (Figure 2A), and a well-demarcated purpuric lesion over her distal right foot, which was cool to touch and did not have a palpable pulse (Figure 2B). Her functional protein C level was <10% and protein S was 41% at the time of diagnosis. Vitamin K and therapeutic fondaparinux were administered intravenously. At this time, the patient was obtunded and unable to participate in shared decision making. The patient's next of kin, her mother, decided to proceed with a comfort focused approach, and the patient died shortly thereafter. 

## Discussion

It is known that warfarin, a vitamin K antagonist, inhibits the synthesis of factors 2, 5, 7, 9, 10, and protein C and S. The inhibition effect on protein C has a faster onset than the other factors, leading to an acute prothrombotic state. The addition of warfarin can lead to augmenting the hypercoagulable state in HIT [3]. The patient’s markedly decreased functional protein C level may have favored the development of WISN.

One hypothesis for the rapid rise in INR is due to underlying vitamin K deficiency given the patient’s malnutrition and antibiotic exposure during her prolonged hospitalization [4,5]. As such, administering warfarin with underlying vitamin K deficiency may have an augmented effect, which in the acute phase is prothrombotic. In this patient’s case, her INR dramatically increased in less than 48 hours. According to current guidelines, the patient was managed appropriately, i.e. the argatroban was stopped, repeat INR was still quite high, and argatroban and warfarin were both held. Additionally, there was an appropriate delay between stopping argatroban and warfarin to account for argatroban’s INR-raising effect [6]. One study demonstrated that the risk of thrombosis outweighs bleeding and cautioned clinicians against reducing or discontinuing argatroban or warfarin, even in the setting of an INR above 4 [7]. However, there are currently no published guidelines stratified by supratherapeutic INR levels or a safe maximum duration off anticoagulation while the INR is supratherapeutic. Thus, current guidelines must be updated to identify a new standard of care for situations with very high INRs.

## Conclusions

HIT and WISN are ingrained in clinicians as serious complications of anticoagulation. Although a rare phenomenon, the likelihood of WISN increases in the setting of hypercoagulable states, such as HIT. Caution should be used when anticoagulating patients with prolonged hospitalizations or other risk factors for vitamin K deficiency. Clinical decision making when treating supratherapeutic INRs in this setting should include any additive effects of direct thrombin inhibitors with vitamin K antagonists. Potential future steps include devising guidelines for very high supratherapeutic INRs when bridging to warfarin and a safe maximum duration off anticoagulation.

## References

[1] Warkentin TE (2003). Heparin-induced thrombocytopenia: pathogenesis and management. Br J Haematol.

[2] Abdul-Jabar HB, Geroulakos G, Philpott N, Fareed J (2006). Warfarin-induced skin necrosis: a case report. Clin Appl Thromb Hemost.

[3] Srinivasan AF, Rice L, Bartholomew JR (2004). Warfarin-induced skin necrosis and venous limb gangrene in the setting of heparin-induced thrombocytopenia. Arch Intern Med.

[4] Alperin JB (1987). Coagulopathy caused by vitamin K deficiency in critically iII, hospitalized patients. JAMA.

[5] Retter A, Barrett NA (2015). The management of abnormal haemostasis in the ICU. Anaesthesia.

[6] Cuker A, Cines DB (2012). How I treat heparin-induced thrombocytopenia. Blood.

[7] Bartholomew JR, Hursting MJ (2005). Transitioning from argatroban to warfarin in heparin-induced thrombocytopenia: an analysis of outcomes in patients with elevated international normalized ratio (INR). J Thromb Thrombolysis.

